# Geriatric distal femoral fractures: post-operative complications and nine-year mortality—a retrospective analysis of two tertiary trauma centres

**DOI:** 10.1007/s00264-023-06075-3

**Published:** 2024-01-04

**Authors:** Camilo A. Delgadillo, Jorge Rojas Lievano, Carlos M. Olarte

**Affiliations:** 1 Universidad del Rosario, School of Medicine and Health Science, Bogota, Colombia; 2 Department of Orthopedics and Traumatology, Hospital Universitario Fundación Santa Fe de Bogota, Bogota, Colombia; 3 School of Medicine, Universidad de Los Andes, Bogota, Colombia; 4 Department of Orthopedics and Traumatology, Hospital Infantil Universitario de San Jose, Bogota, Colombia

**Keywords:** Distal femur fracture, Mortality, Time to surgery, Complication

## Abstract

**Purpose:**

This study aimed to estimate the mortality at various post-operative intervals and explore influential variables for these outcomes in elderly patients with distal femur fractures (DFF).

**Methods:**

A retrospective observational study was conducted across two tertiary care institutions, between 2014 and 2020. The primary outcomes were mortality rates at 30-day, six month, and one year post-surgery. Secondary outcomes included 1-year readmission and reintervention rates along with their correlated complications.

**Results:**

A total of 37 DFF in 35 patients was analyzed; average age was 83.6 years (range, 65–98 years). The overall mortality rate at a maximum follow-up of 8.8 years was 74% (26/35 patients). The median survival time was 3.2 years and the survival probability at five years was 27% (95% confidence interval [CI], 13 to 43%). Mortality rates at 30 days, six months, and one year after surgery were 8.6% (3 patients), 23% (8 patients), and 34% (12 patients), respectively. Overall mortality rate was 64% (15/24 patients) for native distal femur fractures, and 92% (13/14 patients) for periprosthetic fractures (*p* = 0.109). Patients older than 85 years and male gender were identified as risk factors for mortality within the first year post-operatively.

**Conclusion:**

Elderly fractures have a high mortality at eight years of follow-up. Mortality at one year was much higher than in other studies of the same nature. We did not find statistically significant differences when comparing native bone fractures with periprosthetic fractures. Factors that impact mortality were being a man, advanced age, elevated index comorbidity, and dementia. There is no relationship between the time to be taken to the surgical procedure and mortality results.

## Introduction

Geriatric distal femur fractures (DFFs) represent a significant clinical challenge. As our global population ages, the prevalence and impact of these injuries on healthcare systems and patient quality of life are expected to rise [1]. While the anatomical intricacies of the distal femur pose a distinct challenge in terms of surgical management, the often compromised physiological status of the elderly adds another layer of complexity to treatment strategies. Extensive research surrounds hip fractures due to their prevalence and recognized complications [2–5]; however, DFFs are less well understood. Current literature has delved into the incidence, epidemiology, and management modalities of DFFs [1, 5–8]. However, there remains a discernible gap in understanding the post-operative outcomes, especially when comparing native to periprosthetic fractures in the geriatric cohort.

As the trend towards total knee arthroplasty (TKA) continues to grow among the elderly, the incidence of periprosthetic distal femoral fractures (pDFF) is also on the rise [8, 9]. These fractures, owing to their unique nature, present distinct challenges, like comminution and secondary loosening, worsened by associated osteoporosis [10]. Although existing literature on pDFFs has highlighted concerning post-treatment mortality rates of 13 to 17% at one year [7, 11, 12], comprehensive data spanning both native and periprosthetic fractures, along with their comparative outcomes, is sparse. Additionally, the influence of specific variables—ranging from patient demographics and pre-existing conditions to the chosen treatment modalities on patient outcomes—remains a pivotal area of research.

In light of these challenges and the paucity of comprehensive data, our study seeks to provide an in-depth analysis of post-operative outcomes in elderly patients surgically managed for both native and periprosthetic DFFs. By exploring mortality, readmission, reintervention rates, and the factors influencing these outcomes, this research aspires to deliver valuable insights. Our objective is to enable clinicians to optimize management strategies, thus enhancing the quality of care for this vulnerable and expanding patient group.

## Methods

### Study design and patient selection

Following each institutional review board approvals, two collaborating tertiary care trauma centers provided a comprehensive list of patients diagnosed under the ICD-10 code S72- and T84-, which pertains to femur fractures and complications of internal orthopedic prosthetic devices, implants, and grafts, between January 2014 and December 2020. These dates were selected due to the implementation of the orthogeriatric program for hip fractures in both institutions [13]. From this list, radiographs were meticulously reviewed to validate the diagnosis and confirm the presence of both native and periprosthetic distal femur fractures. Inclusion criteria consisted of patients aged 65 years and older who had sustained fractures from low-energy injuries, such as falls from a standing height, who was treated surgically. Patients with fractures resulting from high-energy mechanisms or with other underlying pathological causes, except osteoporosis, were excluded.

### Patient and fracture characteristics

The demographics and clinical data of the selected patients were retrospectively collected from charts including sex, age, body mass index (BMI), and comorbidities. Radiographic evaluations of the femur and knee were undertaken to classify the fractures based on the AO/OTA system [14]. Further, the presence of a total knee arthroplasty (TKA) or a total hip arthroplasty (THA) was identified. For those with periprosthetic fractures, The Unified Classification System (UCS) was employed for categorization [15].

Operative data were also collected, including the time interval between admission and surgery, duration of surgery, volume of intraoperative blood loss, and type of reconstructive technique used. To assess and quantify the patient’s comorbidities at the time of injury, the age-adjusted Charlson Comorbidity Index (CCI) was calculated using both clinical records and ICD-10 codes [16]. The age-adjusted CCI is an enhancement of the original CCI. While the original index quantifies mortality risk by scoring comorbidities based on their potential to impact mortality, the age-adjusted CCI incorporates age into the scoring system, offering a more comprehensive risk assessment by accounting for both comorbidity severity and the patient’s age [17]. Additionally, the Barthel index for Activities of Daily Living (ADL) at the time of injury was collected from patient charts to gain insights into the patient’s functional status pre-injury [18, 19].

### Post-operative variables and outcomes

Post-operative data included transfusion requirements, admission to the intensive care unit (ICU), length of hospital stay, occurrence of medical complications, surgical site infections, reintervention, and readmission. To determine patient mortality, the Unique Affiliate Registry Death Module (RUAF-D, for the Spanish acronym) database, which records all deaths nationally, was referenced. The specific date of death, where applicable, was documented.

### Surgical technique

All surgeries were performed by expert trauma surgeons or arthroplasty surgeons, according to characteristics of each fracture. Specific surgical techniques and implant choices were based on the fracture type, surgeon’s preference, and individual patient factors. Following surgical intervention, patients were allowed immediate full weight-bearing without the need for knee immobilization, if there were no contraindications. This approach was adopted to encourage early mobilization and to capitalize on the stability afforded by modern fixation techniques [20]. Roughly secondary days post-surgery, patients commenced guided physical therapy. This initial phase primarily emphasized restoring range of motion and joint flexibility and mitigating post-operative swelling during in-stay hospitalization. After discharge, assisted home-care physical rehabilitation was introduced.

### Statistical analysis

The data were summarized using means and standard deviations for continuous variables, while counts and percentages were used for categorical variables. The unadjusted cumulative mortality was calculated utilizing the product limit method. To estimate the probability of mortality over time, the Kaplan-Meier survival method was employed. To compare survival curves, log-rank tests were performed. Cox regression was used to assess the effect of the following variables on survivorship: age, gender, age-adjusted CCI, BMI, type of fracture, and time from admission to surgery. The effects of certain factors, like surgical delay, on outcomes such as mortality were analyzed by comparing patients who received surgery within the first 48 h of admission, between 48 and 96 h post-admission, and after four days of admission. A logistic regression model was performed to explore variables associated with one year mortality and reintervention after surgical treatment of DFFs. Significance was assessed at an alpha level of 0.05.

The additional risk per 1000 patients for each significant factor identified in the multivariate analysis was calculated as [(HR−1) × I0 × 1000], where HR represents the hazard ratio for the risk category compared to the reference category, and I0 corresponds to the unadjusted cumulative incidence of mortality for the reference category. All statistical analyses were performed using Stata 14 software (StataCorp, College Station, TX, USA).

## Results

The study included 35 consecutive patients who underwent surgical management of 37 distal femur fractures; two patients sustained bilateral fractures. The average age was 83.6 years (range, 65–98 years), with females representing 31 patients of the cohort. The average age-adjusted CCI was 4.5 (range, 0–8). The average Barthel index for ADLs was 63.8 (range, 0–100) with more than half the patients being minimally dependent or independent (*n* = 21; 60%). All included fractures were closed, and 29 fractures were extraarticular. A total of 14 fractures were periprosthetic fractures constituted (38%). From admission, the median time to surgery was 39 h (range, 3 h–21 days). Lateral locking compression plates were the primary treatment for 24 fractures (65%). Other reconstructions were used less commonly such as double-plating fixation, combination of a retrograde femoral nail and lateral locking plating, and distal femoral replacement (Table 1). Surgery lasted a median of 179 min (range, 90–387 min). Following the procedure, 23 patients (62%) needed at least 1 unit of blood transfusion, and 10 patients (10%) were admitted to the ICU. Median length of stay was seven days, with a span from one to 61 days (Table 1).

**Table 1:** Demographic and baseline characteristics of the patients and operative data*

Characteristic	Non-periprosthetic (*N* = 23)	Periprosthetic (*N* = 14)	All fractures (*N* = 37)	*p* value
Age—years				.198
Mean	82.2 ± 9.1	86.1 ± 8.2	83.6 ± 8.8	
Range	65–98	70–97	65–98	
Sex—no. (%)				.575
Female	20 (87)	13 (93)	33 (89)	
Male	3 (13)	1 (7)	4 (11)	
Body mass index (kg/m^2^)				.899
Mean	26.3 ± 5.1	26.5 ± 5.3	26.3 ± 5.1	
Range	18.7–38.5	15.6–34.3	15.6–38.5	
Age-adjusted CCI				.534
Mean	4.4 ± 1.9	4.8 ± 1.7	4.5 ± 1.8	
Range	1–8	1–7	1–8	
Barthel index for ADLs				.534
Mean	64.7 ± 30.4	62.1 ±28.2	63.8 ± 29.2	
Range	0–100	30–100	0–100	
OTA/AO classification—no. (%)^†﻿^				
33A	14 (61)	-	-	
33B	5 (22)	-	-	
33C	4 (17)	-	-	
UCS classification—no. (%)^††﻿﻿^				
IV.3C (hip, femur, distal to the implant)	-	3 (21)	-	
V.3B (knee, femur, bed of or around the implant)	-	10 (71)	-	
V.3C (knee, femur, proximal to the implant)	-	1 (7.1)	-	
Time from admission to surgery—no. (%)				.017
>48 h	17 (74)	6 (43)	23 (62)	
48–96 h	5 (22)	2 (14)	7 (19)	
>96 h	1 (4.3)	6 (43)	7 (19)	
Operative data				
Surgery duration (minutes)				.273
Median	171	187	179	
Range	90–387	123–365	90–387	
Type of reconstruction—no. (%)				.020
Lateral locking compression plate	18 (78)	6 (43)	24 (65)	
Double-plating fixation	0 (0)	3 (21)	3 (8.1)	
Retrograde femoral nail + lateral locking plate	2 (8.7)	1 (7.1)	3 (8.1)	
Revision prosthesis	0 (0)	1 (7.1)	1 (2.7)	
Distal femoral replacement	0 (0)	3 (21)	3 (8.1)	
External fixator	1 (4.3)	0 (0)	1 (2.7)	
Retrograde femoral nail	2 (8.7)	0 (0)	2 (5.4)	
Postoperative data				
Transfusion of at least 1 unit of RBCs	12 (52)	11 (76)	23 (62)	.108
Postoperative ICU admission	7 (30)	3 (21)	10 (27)	.550
Length of stay (days)				.165
Median	7	7.5	7	
Range	2–42	1–61	1–61	
Reintervention	5 (22)	2 (14)	7 (19)	.575

*Plus–minus values are means ± SD

^†^AO Foundation/Orthopaedic Trauma Association (AO/OTA) fracture classification

^††^Unified Classification System (UCS)

### Mortality

**Fig. 1:**
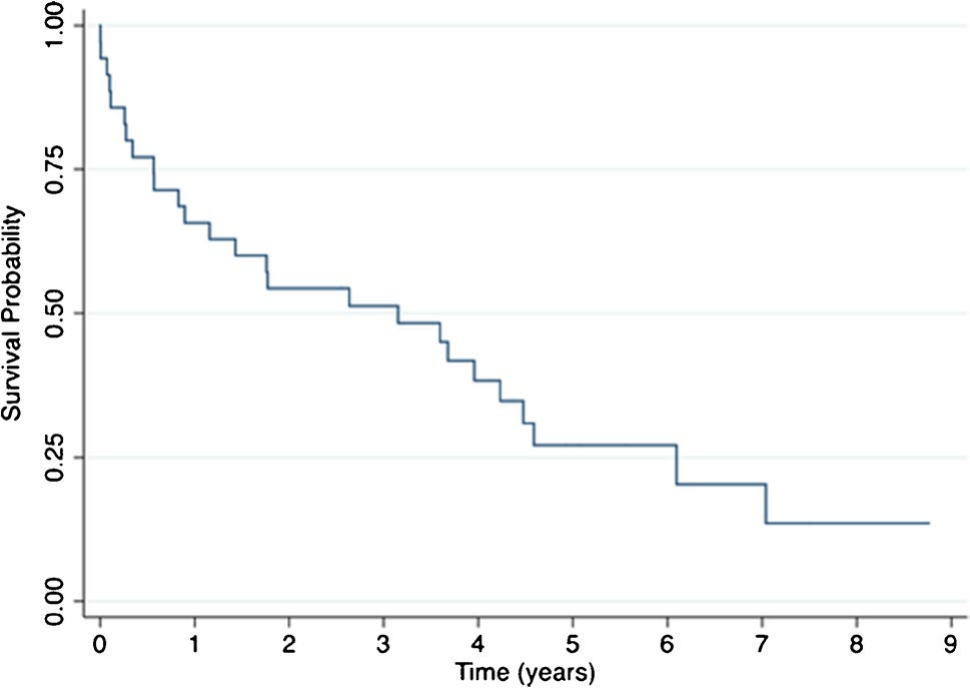
Kaplan-Meier survival curves show survivorship of distal femur fractures for the entire cohort of patients

**Fig. 2:**
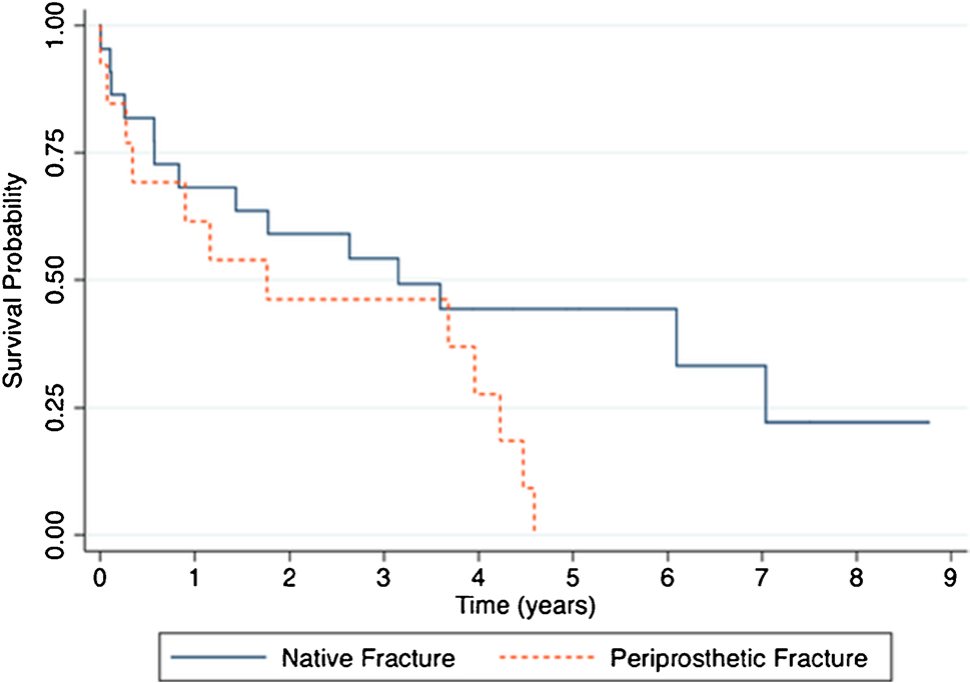
Kaplan-Meier survival curves show the survival of distal femur fractures in the presence or absence of knee or hip arthroplasty

The overall mortality rate at a maximum follow-up of 8.8 years was 26/35 patients (74%). The median survival time was 3.2 years, and the survival probability at five years was 27% (95% confidence interval [CI], 13 to 43%) (Fig. 1). Mortality rates at 30 days, six months, and one year after surgery were three patients (8.6%), eight patients (23%), and 12 patients (34%), respectively. The three patients who died during the first 30 days had an average age of 88.7 years (range, 72–97 years), and an average age-adjusted CCI of 4.5 (range, 4–5). Of these three patients, two died while in the hospital. Overall mortality rate for native distal femur fractures was 15 of 24 patients (64%) and 13 of 14 patients (92%) for periprosthetic fractures (*p* = 0.109). Thirty-day, six month, and one year mortality rates were one patient (4.5%), four patients (18%), and seven patients (32%) for the native fracture group and one patient (5%), four patients (31%), and five patients (38%) for the periprosthetic fracture group (*p* = 0.54, 0.43, and 0.69, respectively). Survival curves comparing native fractures to periprosthetic fractures revealed analogous trends for the initial three years; however, by the fifth post-operative year, the periprosthetic group displayed a pronounced mortality decline, with most patients deceased (log-rank test *p*-value = 0.07) (Fig. 2).

Patients older than 85 years (OR 7.9, 95% CI 1.3–46.9) and male gender (OR 16.8, 95% CI 1.1–253.9) were identified as risk factors for mortality within the first year post-operatively. Cox regression analysis also revealed dementia (HR 3.1, 95% CI 1.03 –9.4), a higher age (HR 1.1, 95% CI 1.06–1.2), and a lower Barthel index (<40 points) (HR 0.97, 95% CI 0.95–0.99) led to shorter survival time. Delay to surgery also was not identified as an independent predictor for mortality.

### Complications

During the first post-operative year, 24 medical complications requiring hospital admissions were documented. Delirium was the most frequent, affecting eight of patients (22%). This was followed by lower respiratory tract infections in five patients (14%) and gastrointestinal bleeding in three patients (8.6%). Other complications included deep vein thrombosis, subdural hematomas from falls and urinary retention in two patients (5.7%) for each, and singular cases (2.9% each) of pulmonary thromboembolism and aspiration pneumonia.

Regarding surgical-related complications, of the 37 procedures, surgical site infection was reported in three cases (8.1%), non-union in three cases (8.1%), and fixation failure in two patients (5.4%). Seven of these complications led to reinterventions, accounting for a 19% reintervention rate. Among the infections, two required early debridement, while the third was managed solely with antibiotics, preserving the implant in all instances. In terms of non-unions, a bone graft paired with a double-plating technique was used in two cases, while the third necessitated a revision knee prosthesis. Among the fixation failures, one was corrected with an added medial femoral plate, and the severity of the other required an above-the-knee amputation. Osteoporosis emerged as the only independent risk factor increasing the likelihood of reintervention in our cohort (OR 7.04, 95% CI 1.01–48.1).

## Discussion

Distal femur fractures (DFFs) in the elderly present a multidimensional clinical challenge. Given their increased incidence in our aging global population, alongside a concurrent surge in total knee and hip arthroplasties, a robust understanding of post-operative outcomes becomes crucial. Our study demonstrated a mortality rate of three patients (8.6%) at 30 days, eight patients (23%) at six months, and 12 patients (34%) at one year among elderly patients treated for DFFs. These findings largely align with the current body of literature, with 30-day mortality rates reported by several researchers ranging from 6 to 9.68% [21–23]. Similarly, for six month and one year rates, our data closely match the literature, which reports values spanning from 17 to 26% and from 13.4 to 38%, respectively [7, 11, 24, 25].

A noteworthy observation from our study is the nuanced differentiation in mortality based on the fracture type, either native or periprosthetic. Although the difference in mortality rates between these groups was not statistically significant, possibly due to our sample size, the declining survival trend in the periprosthetic group post the third year is conspicuous and warrants exploration. This trend possibly underscores the inherent complexities of managing periprosthetic fractures, which are known for their intricate challenges.

In a comparative light, the mortality rates we identified for DFFs seem to eclipse those documented for hip fractures in several investigations, underscoring the serious clinical implications of DFFs [8, 25]. In our Institution’s hip fractures, mortality is 10.9% at one year [13, 26]. Given the traditionally heightened focus on hip fractures in medical discourse and care protocols, the collective insights from our study and pre-existing research might necessitate a reevaluation of clinical perspectives and resource deployment. The pronounced morbidity and mortality tied to DFFs spotlight the necessity for specialized geriatric orthopaedic care. Tsai et al. [8], expounded upon the profound effects of DFFs on patient outcomes, such as extended hospital stays and decreased instances of discharges to home settings. Our findings reinforce the need for targeted interventions and specialized care pathways for this vulnerable patient population. Delving into the root causes, pinpointing interventions to curtail these rates, and exploring long-term morbidity and post-fracture quality of life are essential future directives.

Delving deeper into our findings, variables such as age (>85 years) and gender (male) emerged as prominent determinants for mortality within the first post-operative year. However, this contrasts with findings from Streubel et al. [7], who did not single out age as an isolated predictor. Another significant predictor was dementia, consistent with the general understanding of the morbidity associated with cognitive impairments in the elderly undergoing surgery. Surgical timing, often a critical point of discussion, did not emerge as a predictor for mortality in our cohort. There are case series that show optimal results of intramedullary fixation with closed reduction without intraoperative imaging, reducing operative time [27, 28]. Our results here diverge from Streubel et al. [7] and Myers et al. [11], this disparity can be attributed to diverse patient populations, institutional practices, or even the relative health of the cohorts studied. We posit that optimizing patient health pre-operatively through comprehensive, multidisciplinary care might neutralize the potential detrimental effects of surgical delays. This underscores the value of holistically preparing patients for surgery, especially those with identified risk factors like advanced age, low Barthel scores, and dementia.

Regarding surgical methods, our predominant reliance on lateral condylar support plates and the relatively low rate of endoprosthesis or knee revision prosthesis emphasizes the advances in orthopaedic technology and techniques. As a corollary, even with the challenges of osteoporotic bone—frequently diagnosed pre-operatively in our patients—this condition was the only independent factor for reintervention, but amputation rates were kept at a minimum. This reflects the leaps in implant technology and nuanced surgical decision-making, which has evolved from skeletal tractions and long leg cast to currently use of locking compression plate (LCP) condylar plate fixation and carbon fibre plates (CFR-PEEK) [10]. However, the reintervention rate, standing at seven cases (19%), is a reminder of the persistent challenges posed by DFFs, corroborating findings from other studies [25]. Comparing our findings with those of Hart et al. [29], we find no marked difference in patient mobility and LOS post-surgery, irrespective of the surgical method employed.

Therefore, emphasis should be placed on stable constructs such as a double plate, with reported high union rates and acceptable ranges of motion in both native DFFs and periprosthetic fractures compared with one lateral plate stabilization; also, double plate allows early complete weightbearing [30, 31], which is critical in the treatment of elderly fractures proposed by Kammerlander et al. [32]. A meta-analysis carried out by Koso et al. [33] reported higher rates of non-union in distal femur fractures, which is why there are case series that recommend the use of fibula autografts with double plates to reduce the possibility of non-unions [34].

Several limitations inherent to our study merit acknowledgment. Primarily, the retrospective design introduces the potential for biases, notably selection bias. While we undertook a meticulous review of medical records, the nature of chart reviews can sometimes lead to overlooking or misclassification of specific data points. The sample size, being relatively small, particularly when considering subgroups such as periprosthetic versus native DFFs, might limit the statistical power of our analyses, potentially masking subtle yet clinically significant differences. While osteoporosis was recognized as a pivotal factor, we did not undertake a comprehensive evaluation of bone quality or delve into osteoporotic severity, which might have nuanced implications for outcomes. Despite these constraints, our study offers valuable perspectives on DFF management in the elderly, and future research could address these limitations with larger, more diverse, and prospective study designs.

## Conclusions

Our study highlights the profound impact of DFFs on the elderly population. The observed one year mortality rate was 34%, escalating to a notable 74% over an 8.8-year follow-up. Especially concerning was the pronounced decline in survival for those with periprosthetic fractures by the fifth year. Age, male gender, dementia, and a lower Barthel index were significant determinants of mortality. Furthermore, osteoporosis was identified as a pivotal factor leading to reinterventions. Contrary to some expectations, delay to surgery did not independently predict mortality. The severity of these outcomes emphasizes the imperative for tailored care strategies for this vulnerable demographic.

## Declarations

This retrospective chart review study involving human participants was in accordance with the ethical standards of the institutional and national research committee and with the 1964 Helsinki Declaration and its later amendments or comparable ethical standards. The Human Investigation Committee of Fundacion Santa Fe de Bogota and Hospital San Jose Infantil approved our study.

## References

[1] Canton G, Giraldi G, Dussi M, Ratti C, Murena L (2009). Osteoporotic distal femur fractures in the elderly: peculiarities and treatment strategies. Acta Biomed.

[2] Martinet O, Cordey J, Harder Y, Maier A, Bühler M, Barraud GE (2000). The epidemiology of fractures of the distal femur. Injury.

[3] Uzoigwe CE, Burnand HGF, Cheesman CL, Aghedo DO, Faizi M, Middleton RG (2013). Early and ultraearly surgery in hip fracture patients improves survival. Injury.

[4] Johnell O, Kanis JA (2004). An estimate of the worldwide prevalence, mortality and disability associated with hip fracture. Osteoporos Int.

[5] Haentjens P, Magaziner J, Colón-Emeric CS, Vanderschueren D, Milisen K, Velkeniers B (2010). Meta-analysis: excess mortality after hip fracture among older women and men. Ann Intern Med.

[6] Pagenkopf E, Grose A, Partal G, Helfet DL (2006). Acetabular fractures in the elderly: treatment recommendations. HSS J.

[7] Streubel PN, Ricci WM, Wong A, Gardner MJ (2011). Mortality after distal femur fractures in elderly patients. Clin Orthop Relat Res.

[8] Tsai SHL, Lin TY, Tischler EH, Hung KH, Chen CH, Osgood GM (2021). Distal femur fractures have a higher mortality rate compared to hip fractures among the elderly: insights from the National Trauma Data Bank. Injury.

[9] Moloney GB, Pan T, Van Eck CF, Patel D, Tarkin I (2016). Geriatric distal femur fracture: are we underestimating the rate of local and systemic complications?. Injury.

[10] Nester M, Borrelli J (2023). Distal femur fractures management and evolution in the last century. Int Orthop.

[11] Myers P, Laboe P, Johnson KJ, Fredericks PD, Crichlow RJ, Maar DC (2018). Patient mortality in geriatric distal femur fractures. J Orthop Trauma.

[12] Dombrowski ME, O’Malley MJ (2018). Indications for distal femoral replacement arthroplasty in acute geriatric distal femoral fractures. Oper Tech Orthop.

[13] Suarez S, Pesantez RF, Diaz ME, Sanchez D, Tristancho LJ, Vanegas MV (2017). Impact on hip fracture mortality after the establishment of an orthogeriatric care program in a Colombian hospital. J Aging Health.

[14] Meinberg E, Agel J, Roberts C (2018). Fracture and dislocation compendium—2018. JOT.

[15] Duncan C, Haddad F (2014). The Unified Classification System (UCS): improving our understanding of periprosthetic fractures. Bone Jt J.

[16] Charlson M, Pompei P, Ales K (1987). A new method of classifying prognostic comorbidity in longitudinal studies: development and validation. J Chronic Dis.

[17] Zhang XM, Wu XJ, Cao J, Guo N, Bo HX, Ma YF (2009). Effect of the age-adjusted Charlson comorbidity index on all-cause mortality and readmission in older surgical patients: a national multicenter, prospective cohort study. Front Med.

[18] Uchinaka E, Hanaki T, Morimoto M, Murakami Y, Tomoyuki M, Yamamoto M (2022). The Barthel index for predicting postoperative complications in elderly patients undergoing abdominal surgery: a prospective single-center study. In Vivo (Brooklyn).

[19] Tomita Y, Yamamoto N, Inoue T, Ichinose A, Noda T, Kawasaki K (2022). Preoperative and perioperative factors are related to the early postoperative Barthel Index score in patients with trochanteric fracture. Int J Rehabil Res.

[20] Consigliere P, Iliopoulos E, Ads T, Trompeter A (2019). Early versus delayed weight bearing after surgical fixation of distal femur fractures: a non-randomized comparative study. Eur J Orthop Surg Traumatol.

[21] Butt MS, Krikler SJ, Ali MS (1996). Displaced fractures of the distal femur in elderly patients: operative versus non-operative treatment. J Bone Jt Surg - Ser B.

[22] Wolf O, Mukka S, Ekelund J, Möller M, Hailer N (2021). How deadly is a fracture distal to the hip in the elderly? An observational cohort study of 11,799 femoral fractures in the Swedish Fracture Register. Acta Orthop.

[23] Mubarak I, Abouelela A, Genena A, Al Ghunimat A, Sarhan I, Ashwood N (2020). Mortality following distal femur fractures versus proximal femur fractures in elderly population: the impact of best practice tariff. Cureus.

[24] Dunlop D, Brenkel J (1999). The supracondylar intramedullary nail in elderly patients with distal femoral fractures. Injury.

[25] Jordan RW, Chahal GS, Davies M, Srinivas K (2014) A comparison of mortality following distal femoral fractures and hip fractures in an elderly population. Adv Orthop Surg. 10.1155/2014/873785

[26] Olarte CM, Zuluaga M, Guzman A, Camacho J, Lasalvia P, Garzon N et al (2021) Analysis of the experience of the geriatric fracture program in two institutions in Colombia: a reproducible model. Colomb Med 52(3). 10.25100/cm.v52i3.4524 10.25100/cm.v52i3.4524 PMC8973310 35431358

[27] Bombah FM, Lékina FA, Eone DH, Dakouré PWH, Sermon A (2022). Focus on interlocking intramedullary nailing without fluoroscopy in resource-limited settings: strategies, outcomes, and outlook. Int Orthop.

[28] Adesina SA, Eyasan SU, Amole IO, Awotunde OT, Akinwumi AI, Durodola AO (2022). Closed reduction and locked intramedullary nailing of diaphyseal long bone fractures without intra-operative imaging and fracture table. Int Orthop.

[29] Hart G, Kneisl J, Springer B, Patt J (2017). Open reduction vs distal femoral replacement arthroplasty for comminuted distal femur fractures in the patients 70 years and older. J Arthroplast.

[30] Seo JH, Lee BS, Kim JM, Kim JJ, Kim JW (2022). Outcomes of dual plating for unstable distal femoral fractures: a subgroup comparison between periprosthetic and non-periprosthetic fractures. Int Orthop.

[31] Nam DJ, Kim MS, Kim TH, Kim MW, Kweon SH (2022). Fractures of the distal femur in elderly patients: retrospective analysis of a case series treated with single or double plate. J Orthop Surg Res.

[32] Kammerlander C, Pfeufer D, Lisitano L, Mehaffey S, Böcker W, Neuerburg C (2018). Inability of older adult patients with hip fracture to maintain postoperative weight-bearing restrictions. J Bone Jt Surg.

[33] Koso R, Terhoeve C, Steen R, Zura R (2018). Healing, nonunion, and re-operation after internal fixation of diaphyseal and distal femoral fractures: a systematic review and meta-analysis. Int Orthop.

[34] Ibrahim FM, Ghazawy AKE, Hussien MA (2022). Primary fibular grafting combined with double plating in distal femur fractures in elderly patients. Int Orthop.

